# Establishment and characterization of patient-derived cancer models of malignant peripheral nerve sheath tumors

**DOI:** 10.1186/s12935-020-1128-z

**Published:** 2020-02-19

**Authors:** Rieko Oyama, Fusako Kito, Mami Takahashi, Emi Hattori, Rei Noguchi, Yoko Takai, Marimu Sakumoto, Zhiwei Qiao, Shunichi Toki, Masato Sugawara, Yoshikazu Tanzawa, Eisuke Kobayashi, Fumihiko Nakatani, Shintaro Iwata, Akihiko Yoshida, Akira Kawai, Tadashi Kondo

**Affiliations:** 1 Department of Innovative Seeds Evaluation, National Cancer Center Research Institute, 5-1-1 Tsukiji, Chuo-ku, Tokyo, 104-0045 Japan; 2 Central Animal Division, National Cancer Center Research Institute, 5-1-1 Tsukiji, Chuo-ku, Tokyo, 104-0045 Japan; 3 Division of Rare Cancer Research, National Cancer Center Research Institute, 5-1-1 Tsukiji, Chuo-ku, Tokyo, 104-0045 Japan; 4 Division of Musculoskeletal Oncology, National Cancer Center Hospital, 5-1-1 Tsukiji, Chuo-ku, Tokyo, 104-0045 Japan; 5 Department of Pathology and Clinical Laboratories, National Cancer Center Hospital, 5-1-1 Tsukiji, Chuo-ku, Tokyo, 104-0045 Japan

**Keywords:** Malignant peripheral nerve sheath tumor, Xenograft, Primary culture, Drug screening

## Abstract

**Background:**

Malignant peripheral nerve sheath tumors (MPNSTs) are a rare subtype of soft-tissue sarcoma, derived from a peripheral branch or the sheath of the sciatic nerve, brachial plexus, or sacral plexus. The clinical outcomes for MPNST patients with unresectable or metastatic tumors are dismal, and novel therapeutic strategies are required. Although patient-derived cancer cell lines are vital for basic research and preclinical studies, few MPNST cell lines are available from public cell banks. Therefore, the aim of this study was to establish cancer cell lines derived from MPNST patients.

**Methods:**

We used tumor tissues from five patients with MPNSTs, including one derived from a rare bone tissue MPNST. The tumor tissues were obtained at the time of surgery and were immediately processed to establish cell lines. A patient-derived xenograft was also established when a sufficient amount of tumor tissue was available. The characterization of established cells was performed with respect to cell proliferation, spheroid formation, and invasion. The mutation status of actionable genes was monitored by NCC Oncopanel, by which the mutation of 114 genes was assessed by next-generation sequencing. The response to anti-cancer agents, including anti-cancer drugs approved for the treatment of other malignancies was investigated in the established cell lines.

**Results:**

We established five cell lines (NCC-MPNST1-C1, NCC-MPNST2-C1, NCC-MPNST3-C1, NCC-MPNST4-C1, and NCC-MPNST5-C1) from the original tumors, and also established patient-derived xenografts (PDXs) from which one cell line (NCC-MPNST3-X2-C1) was produced. The established MPNST cell lines proliferated continuously and formed spheroids while exhibiting distinct invasion abilities. The cell lines had typical mutations in the actionable genes, and the mutation profiles differed among the cell lines. The responsiveness to examined anti-cancer agents differed among cell lines; while the presence of an actionable gene mutation did not correspond with the response to the anticipated anti-cancer agents.

**Conclusion:**

The established cell lines exhibit various characteristics, including proliferation and invasion potential. In addition, they had different mutation profiles and response to the anti-cancer agents. These observations suggest that the established cell lines will be useful for future research on MPNSTs.

## Background

Malignant peripheral nerve sheath tumor (MPNST) is a rare subtype of soft-tissue sarcomas representing approximately 10% of all soft-tissue sarcomas [1]. MPNSTs originate from a peripheral nerve branch or the sheath of the sciatic nerve, brachial plexus, or sacral plexus. The emergence of MPNSTs is often associated with neurofibromatosis type 1 (NF1), which is an autosomal dominant disorder characterized by plexiform neurofibroma [2, 3]. MPNSTs occur in approximately 5–42% of patients with NF1, with an incidence of 0.001% in the general population [4]. Half of all MPNST cases develop in individuals with NF1; furthermore, MPNST is the leading cause of death among NF1 patients [5]. The majority of patients with MPNSTs had NF1, suggesting that NF1 is a significant risk factor for MPNST [6]. Mutations in cancer driver genes such as *TP53* [7] and *CDKN2A* [8] are observed in MPNSTs, which do not exhibit chromosomal translocations and display complex genome rearrangements. Moreover, next-generation sequencing analysis has revealed recurrent inactivation mutations in *SUZ12* and *EED*, which are subunits of the Polycomb repressive complex 2 (PRC2) [9]. The loss of RPC2 function may promote Ras pathway activation in MPNST through transcriptional regulatory effects [9–12]. In fact, aberrant regulation of the Wnt/β-catenin [13], PI3K/AKT/mTOR [14], and Raf/MEK/ERK pathways [15] was reported in MPNST. Though the clinical applications of these molecular insights pertaining to MPNST are intriguing, the treatment modalities for MPNST have not been considerably improved, and the disease outcome has not changed significantly in the past several decades [16]. Currently, surgical resection with wide negative margins remains the only curative treatment for MPNST [17]; however, surgery is not always feasible due to tumor location, size, and metastases. Furthermore, the therapeutic utility of radiotherapy and chemotherapy for MPNST has not been established, while clinical trials of investigational agents such as erlotinib [18], sorafenib [19], imatinib [20], dasatinib [21], and alisertib [22] have not successfully demonstrated the efficacy of these drugs. Presently, the clinical outcome for MPNST patients with unresectable or metastatic tumors is dismal [23, 24], and the 5-year survival rate remains approximately 20–50%. Therefore, the development of novel systematic treatments is crucial for improving the quality of life and prognosis of patients with MPNST.

Patient-derived cancer models such as cell lines and xenografts are indispensable tools for furthering our understanding of the molecular mechanisms of carcinogenesis and cancer progression. We can investigate the functions of novel genes and proteins and determine the efficacy of novel drugs using cell lines. Furthermore, cell lines allow high-throughput screening of the anti-tumor effects of investigational drugs [25] and analysis of the impact of gene silencing using CRISPR-Cas9 [26]. According to the largest cell line database, Cellosaurus (Version 28, https://web.expasy.org/cellosaurus/), 24 MPNSTs cell lines have been reported in previous studies. However, only five are available from a public cell bank such as American Type Culture Collection (ATCC) [27, 28]. Considering the above-mentioned heterogeneity of the disease, additional cell lines are required to understand the clinical diversity and malignant characteristics of MPNSTs. Hence, the lack of effective treatments may be attributable to the paucity of proper cancer models for studying MPNSTs.

In this study, we established six patient-derived MPNST cell lines from five patients who exhibited unique clinical features and investigated the morphological and functional characteristics as well as anti-cancer drug responses of these established cell lines.

## Methods

### Patient information

This study included tumor tissues from five patients with MPNST, who underwent surgical resection at the National Cancer Center Hospital, Tokyo, Japan. This study was approved by the ethical committee of the National Cancer Center, and all patients in this study provided written informed consent.

### Establishment of patient-derived xenograft (PDX) models of MPNST

MPNST tissues were obtained at the time of surgery and were subcutaneously grafted into the bilateral hind flanks of 6–12-week-old female mice with a severe immunodeficient phenotype (NOD Cg-*Prkdc*^*scid*^ *Il2rg*^*tm1Sug*^*/Jic,* also known as NOD/Shi-*scid IL*-*2Rγ*^*null*^ or NOG; Central Institute for Experimental Animals, Kanagawa, Japan) using a 13-gauge transplant needle. Tumor size was measured using a digital caliper (SuperCaliper, Mitutoyo, Kanagawa, Japan) and tumor volume was calculated as pi/6 × length × width × thickness [29]. Each tumor was transplanted into another mouse when the tumor volume reached 500–1000 mm^3^. After two passages, the tumors were cryopreserved using Cell Banker 1 plus (Takara Bio, Shiga, Japan) in liquid nitrogen. All animal experiments were performed in accordance with the guidelines for Animal Experiments of the National Cancer Center and approved by the Institutional Committee for Ethics of Animal Experimentation.

### Histological observation

Histological examinations were performed on tumor tissues that were sectioned into 4-µm-thick slices from a representative paraffin-embedded block of the tumor. Then, the tissue sections were deparaffinized and stained with hematoxylin and eosin (HE).

### Primary tissue culture

The tissues of original tumors and PDXs were minced with scissors and passed through an 18-gauge needle. Cell aggregates and tissue fragments were removed with a 70-μm nylon mesh (BD Falcon). Cells were collected by centrifugation for 5 min at 200 x*g*, and seeded in tissue culture plates (Thermo Fisher Scientific, Waltham, MA, USA). The cells were maintained in Dulbecco’s modified Eagle’s medium (DMEM) (Sigma-Aldrich, St. Louis, MO, USA) supplemented with 10% heat-inactivated fetal bovine serum (FBS; Gibco, Grand Island, NY, USA), 100 U penicillin G, and 100 µg/mL streptomycin (Gibco) at 37 °C in a humidified atmosphere of 5% CO_2_. The tissue culture medium was refreshed every several days, and when the cell monolayers reached sub-confluence, the cells were dispersed with 0.1% trypsin–EDTA (Gibco) and seeded into other culture plates.

### Authentication and quality control of the established cell lines

The cell lines were authenticated by analyzing short tandem repeats (STRs) in ten loci using the GenePrint 10 system (Promega, Madison, WI, USA) according to the manufacturer’s instructions. In brief, genomic DNA was extracted from tumor tissues or cultured cells using AllPrep DNA/RNA mini kits (Qiagen, Hilden, Germany), quantified using a NanoDrop 8000 instrument (Thermo Fisher Scientific), and stored at − 80 °C until further use. Genomic DNA (500 pg) was amplified and examined using a 3500xL Genetic Analyzer (Applied Biosystems, Waltham, MA, USA). The digital data were analyzed using the GeneMapper software (Applied Biosystems), and the results were compared with the STR profiles available in public cell banks such as ATCC, Deutsche Sammlung von Mikroorganismen und Zellkulturen (DSMZ), and Japanese Collection of Research Bioresources (JCRB).

Possible contamination with *Mycoplasma* in the established cell lines was also determined. Briefly, DNA was recovered from culture supernatants when the cells reached 70–90% confluence, heated at 95 °C for 10 min, and amplified using the e-Myco *Mycoplasma* PCR detection kit (Intron Biotechnology, Gyeonggi-do, Korea). The amplified DNA was separated using electrophoresis on 1.5% agarose gels, stained with the Midori Green advanced stain (Nippon Genetics, Tokyo, Japan), and analyzed using an Amersham Imager 600 (GE Healthcare Biosciences, Little Chalfont, UK).

### Cell growth analysis

MPNST cells were seeded into 96-well culture plates at densities of 2, 4, and 8 × 10^3^ cells per well (NCC-MPNST1-C1, NCC-MPNST2-C1, NCC-MPNST4-C1, NCC-MPNST5-C1 lines) or 12 and 16 × 10^3^ cells per well (NCC-MPNST3-C1 and NCC-MPNST3-X2-C1 lines), incubated at 37 °C, and analyzed for growth at 24, 48, 72, and 96 h. At the end of each time point, the cell counting kit (CCK)-8 reagent (Dojindo Molecular Technologies, Inc., Kumamoto, Japan) was added to the cells and incubated for 2 h, and the absorbance at 450 nm was measured using a microplate reader (Bio-Rad, Hercules, CA, USA). Growth curves were constructed by plotting the absorbance value (y-axis) versus culture time (x-axis) and were used to calculate the doubling time for each cell line. All experiments were performed in triplicate.

### Spheroid formation assay

To explore spheroid formation, 1 × 10^5^ cells for NCC-MPNST1-C1, NCC-MPNST2-C1, NCC-MPNST3-C1, NCC-MPNST3-X2-C1, and NCC-MPNST4-C1, and 1 × 10^6^ cells for NCC-MPNST5-C1 were seeded into 6-cm plates (Ultra Low Culture Dish; Thermo Fisher Scientific) in DMEM containing 10% FBS and incubated in a humidified atmosphere of 5% CO_2_ at 37 °C. The presence of spheroids was confirmed by microscopic observation (Keyence, Osaka, Japan). All assays were performed in duplicate.

### Transwell cell invasion assay

The invasive ability of the cultured cells was assessed using the Transwell assay and BD Biocoat Matrigel invasion chambers (BD Biosciences, Bedford, MA, USA) according to the manufacturer’s instructions. In brief, 1 × 10^5^ cells were plated in the upper chamber in serum-free DMEM, whereas the medium in the bottom chamber contained 10% FBS, and the plates were incubated for 48 h. The tumor cells invading the bottom surface were stained with Diff-Quick staining kit (Sysmex, Hyogo, Japan) according to the instruction manual, and their number was counted in nine separate areas under a microscope at the magnification of 200×.

### Real-time cell analyzer (RTCA) invasion assay

RTCA Cim-16 plates (xCELLigence Roche, Penzberg, Germany) in a label-free real-time setting was used for RTCA invasion assays according to the manufacturer’s protocol. In brief, Matrigel at a protein concentration of 8.95 mg/mL (BD Biosciences, MA, USA) was subjected to the upper sides of the RTCA membrane, and 4 × 10^4^ cells were seeded into the RTCA upper chamber. The cells were starved in serum-free medium for 24 h. Then, the complete growth medium was added to the RTCA lower chambers as a chemo-attractant. The cells invading from the upper chamber through the Matrigel membrane into the bottom chamber contacted and adhered to the electronic sensors on the underside of the membrane. The attached cells affected the electrical impedance of the membrane; the impedance correlated positively with the number of cells on the bottom of the membrane [30]. The impedance was monitored every 15 min for 126 h.

### Target sequencing with next-generation sequencing-based multiplex gene assay

Alterations, including mutations, amplifications, and homozygous deletions in the coding region of 114 genes (Additional file 1: Table S1) were examined with the NCC Oncopanel test (version 4)—which is a hybridization capture-based next-generation sequencing (NGS) assay—according to the protocol suggested in a previous report [31]. Genomic DNA was extracted from tumor tissues and cell lines using DNeasy Blood & Tissue kit (Qiagen, Hilden, Germany). The extracted DNA was quantified with a nanodrop and Qubit ds DNA BR Assay Kit (Thermo Fisher Scientific) and Qubit 3.0 Fluorometer (Thermo Fisher Scientific). Sequencing libraries were prepared from the extracted DNA (ranging from 50–800 ng) and a KAPA Hyper Prep Kit (KAPA Biosystems, Wilmington, MA, USA) and then sequenced on the Illumina NextSeq (Illumina, San Diego, CA, USA) with 150 bp paired-end reads.

### Screening the anti-proliferative effects of chemotherapeutic drugs

Cells were seeded in a 384-well plate at 5 × 10^3^ cells/well in DMEM supplemented with 10% FBS and incubated at 37 °C in a humidified atmosphere of 5% CO_2_. The following day, the cells were treated with various concentrations of anti-cancer agents (Selleck Chemicals, Houston, TX, USA) for 72 h and analyzed for survival using the CCK-8 reagent according to the manufacturer’s protocol. The list of anti-cancer agents is provided in Additional file 2: Table S2.

### Data analysis

Mapping of NGS reads to the human reference genome was performed with the Burrows-Wheeler Aligner [32] and the Burrows-Wheeler Aligner-Smith-Waterman algorithm [33] after removing adapter sequences with a Cutadapt program. Alterations including single nucleotide variants (SNV), short insertions, and deletions (indels), gene amplifications, homozygous deletions, and gene fusions were detected with cisCall program (version 7.1.7) [34]. SNVs with 5% or more variant allele frequencies and amplifications with more than four copies were defined as positive. Genes with less than 0.5-fold copy decreases were identified as homozygous deletion. Truncating mutations and mutations marked as “pathogenic” in the ClinVar database (20150627) [35] and those reported in the COSMIC database (version 71) [36] were classified as “deleterious”.

Cluster analysis was performed using the R software version 3.4.0 [37]. The distance matrix was determined using the dist function with method = “euclidean.” Hierarchical clustering was achieved using the hclust function with method = “ward.D.” Heat maps were visualized using the “gplots” package of R using the script “heatmap.2.”

## Results

### Overview of workflow

**Fig. 1:**
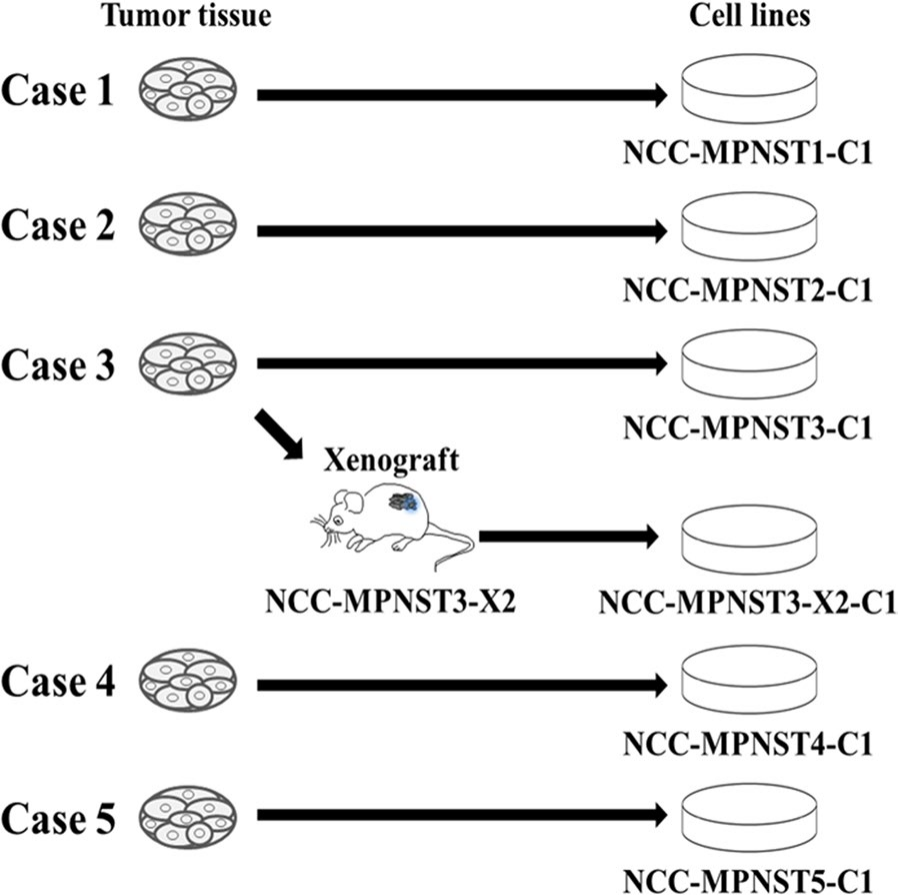
Overall workflow of the study. The cell lines were established from the original tumor tissues of MPNST patients and MPNST-derived PDXs. The names of the established cell lines are indicated below the images of cell cultures

Cell lines were established from tumor tissues of five patients with MPNSTs. The overall design of the study, including cell line designation, is illustrated in Fig. 1.

### Clinical characteristics of the donors

This study included five patients to establish the patient-derived cancer models. The clinical characteristics of the donor patients are presented below. Cell lines as well as their corresponding donor patients and samples are summarized in Table 1.

**Table 1:** Cell lines and their origins

Cell line name	Original case	Original sample
NCC-MPNST1-C1	Case 1	Original tumor tissue
NCC-MPNST2-C1	Case 2	Original tumor tissue
NCC-MPNST3-C1	Case 3	Original tumor tissue
NCC-MPNST3-X2-C1	Case 3	Tumor tissue of second generation xenograft of case 3
NCC-MPNST4-C1	Case 4	Original tumor tissue
NCC-MPNST1-C1	Case 5	Original tumor tissue

**Fig. 2:**
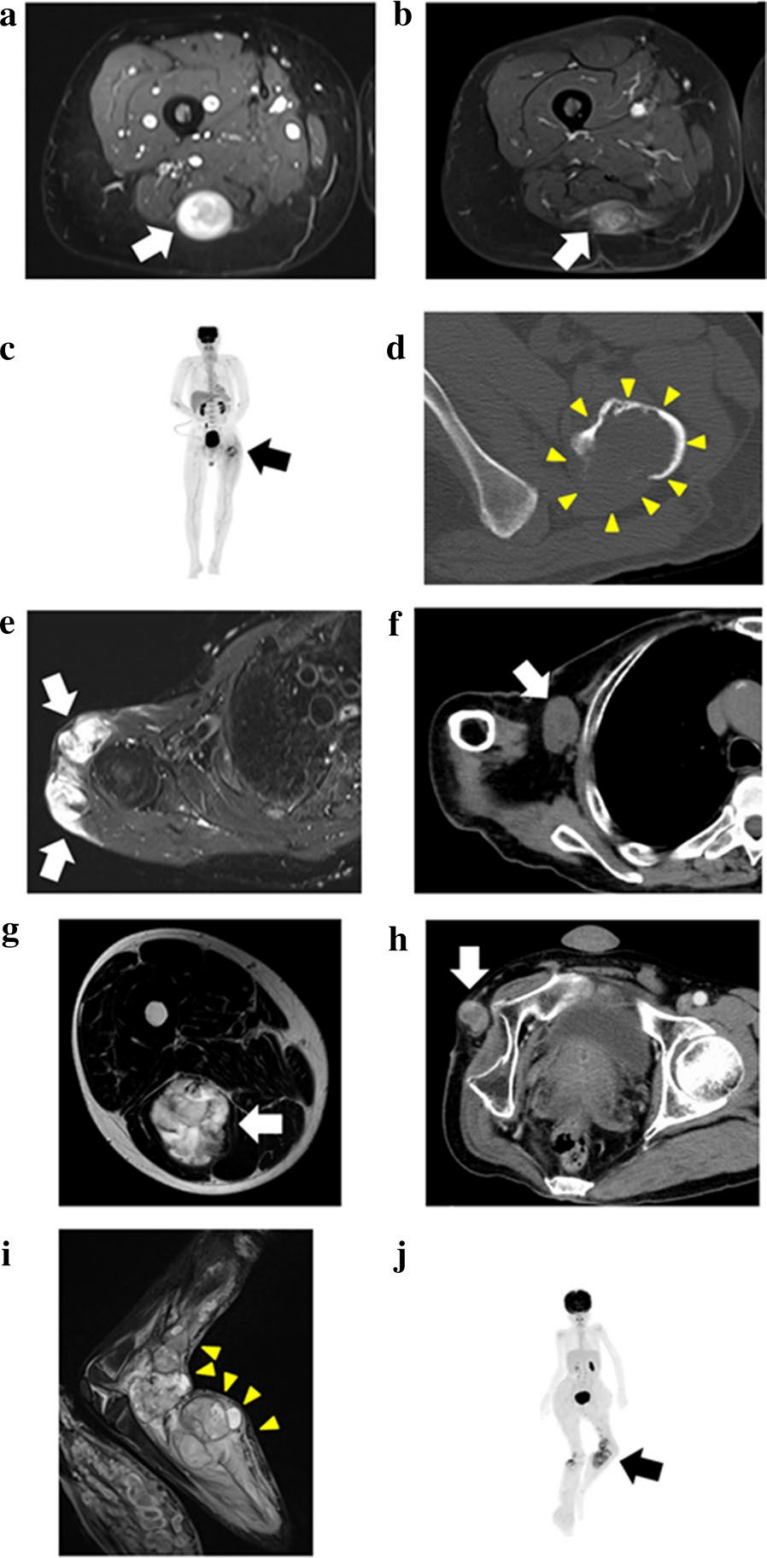
Clinical imaging data of donor patients. **a**, **b** Case 1. T2-weighted short tau inversion recovery sequences of MRI showing a high-intensity lesion with the slightly heterologous interior in the posterior of the right thigh (**a**, *arrow*). Residual rosary-like tumors along the sciatic nerve were observed using gadolinium-enhanced fat-suppressed (MR) imaging (**b**, *arrow*). **c**, **d** Case 2. Positron emission tomography of the whole body showing a bone tumor with a pathological fracture in the left femur and no other oncological lesions (**c**, *arrow*). An axial view of the CT scan at the lesser trochanter level showing a lytic bone tumor in the left femur, which had irregular structure and lacked a cortex, and an extraskeletal mass in the posterior part (**d**, *yellow arrowheads*). **e–f** Case 3. Axial view of the T2-weighted short tau inversion recovery MRI sequences showing a primary soft tissue tumor of the right shoulder documented in the previous hospital (**e**, *arrows*). The lesion, which showed high-intensity signal, infiltrated the surrounding region. Computed tomography images detected a right axillary mass indicative of metastasis (**f**, *arrow*). **g**–**h** Case 4. T2-weighted MRI showing the primary soft tissue tumor as a high-intensity lesion at the posterior aspect of the right thigh (**g**, *arrow*). A recurrent tumor at the stump of the right hip disarticulation, from which the sample for the PDX model was obtained (**h**, *arrow*). **i**–**j** Case 5. Short TI inversion recovery imaging in MRI detected the multi-nodular and heterogeneous intensity signal tumor at the left popliteal fossa (**i**, *yellow arrowheads*). Positron emission tomography-computed tomography of the whole body showed the abnormal uptake at the tumor with SUVmax of 5.81 (**j**, *arrow*)
- Case 2: The donor was a 54-year-old man without NF1. He presented a left femoral bone tumor with a pathological fracture (Fig. 2c, d). The specimen was obtained during the wide excision of the tumor. The patient developed multiple lung and bone metastases in 3 months after the operation and was alive for 7 months after surgery. The details of this case have been previously reported [38]. The cell line established from the tumor tissue was labeled NCC-MPNST2-C1 and was maintained for 50 passages over 36 months.
- Case 3: The donor was a 72-year-old man with NF1 with an original tumor developed in the right shoulder, and several rounds of surgery and radiation therapy had been performed in another hospital. The patient visited the National Cancer Center Hospital for the treatment of multiple metastatic tumors in the sacrum, acetabulum, retroperitoneum, and axilla. A cell line and PDX were created from the resected right axillary lesion, which might be within the radiation field of the original right shoulder MPNST, and designated as NCC-MPNST3-C1 and NCC-MPNST3, respectively. A cell line established from the second-generation PDX (NCC-MPNST3-X2) was designated as NCC-MPNST3-X2-C1. The patient had a local recurrence after 3 months, and progression of other metastatic lesions was observed in the final follow-up 13 months after the surgery despite chemotherapy with doxorubicin and pazopanib; representative MRI and CT images are shown in Fig. 2e, f, respectively. The cell lines were maintained for 20 passages over 20 months and for ten passages over 16 months, respectively.
- Case 4: The donor was a 48-year-old man without NF1. The original tumor developed in the posterior aspect of the right thigh (Fig. 2g). The patient had several local recurrences and re-excisions and was treated with multiple cycles of adjuvant chemotherapy with doxorubicin, ifosfamide, gemcitabine, and docetaxel; 3 weeks after the systematic chemotherapy, he underwent hip disarticulation. The sample used for the cell lines was obtained after resection of the recurrent tumor at the stump of disarticulation (Fig. 2h). The patient was alive with the disease for 2 years and 8 months postoperatively; he had multiple metastases into the lungs, bones, adrenal glands, and soft tissues. The cell line established from the tumor tissue was designated NCC-MPNST4-C1. The cells were maintained for eight passages over 8 months.
- Case 5: The donor was a 9-year-old girl with NF1 who had wide bilateral excisions in her thigh after two surgeries. The surgical margin of the left thigh was positive, and radiotherapy of 50 Gy was performed postoperatively. Almost 1 year later, a rapidly growing mass at the left popliteal fossa was detected using MRI and positron emission tomography-computed tomography (Fig. 2i, j), and she was referred to the National Cancer Center Hospital, Tokyo, Japan. Her leg was amputated above the knee, from which the sample used for establishing the cell line and models for the present study were obtained. This sample represented an MPNST with heterologous rhabdomyoblastic differentiation arising in association with neurofibroma. Two months after the third operation, the patient suffered another wide excision due to MPNST on the right lower leg. At the final observation 1.5 years after the surgery, the patient showed no evidence of malignancy.

- Case 1: The donor was a 37-year-old woman with NF1, who had a first-degree relative with NF1. She had a painful mass on the right posterior thigh detected using magnetic resonance imaging (MRI) (Fig. 2a), which had been increasing in size for a few years. After the unplanned excision of the tumor in another hospital, histological analysis revealed MPNST and residual tumors (Fig. 2b). The patient underwent additional surgery and wide excision of the residual tumors, which were used to establish a cell line. The patient showed no evidence of the disease for 2 years and 8 months after the surgery. The cell line established from the tumor tissue was labeled as NCC-MPNST1-C1 and was maintained for 80 passages over 38 months.

### Growth and morphological characteristics of PDX tumors

**Fig. 3:**
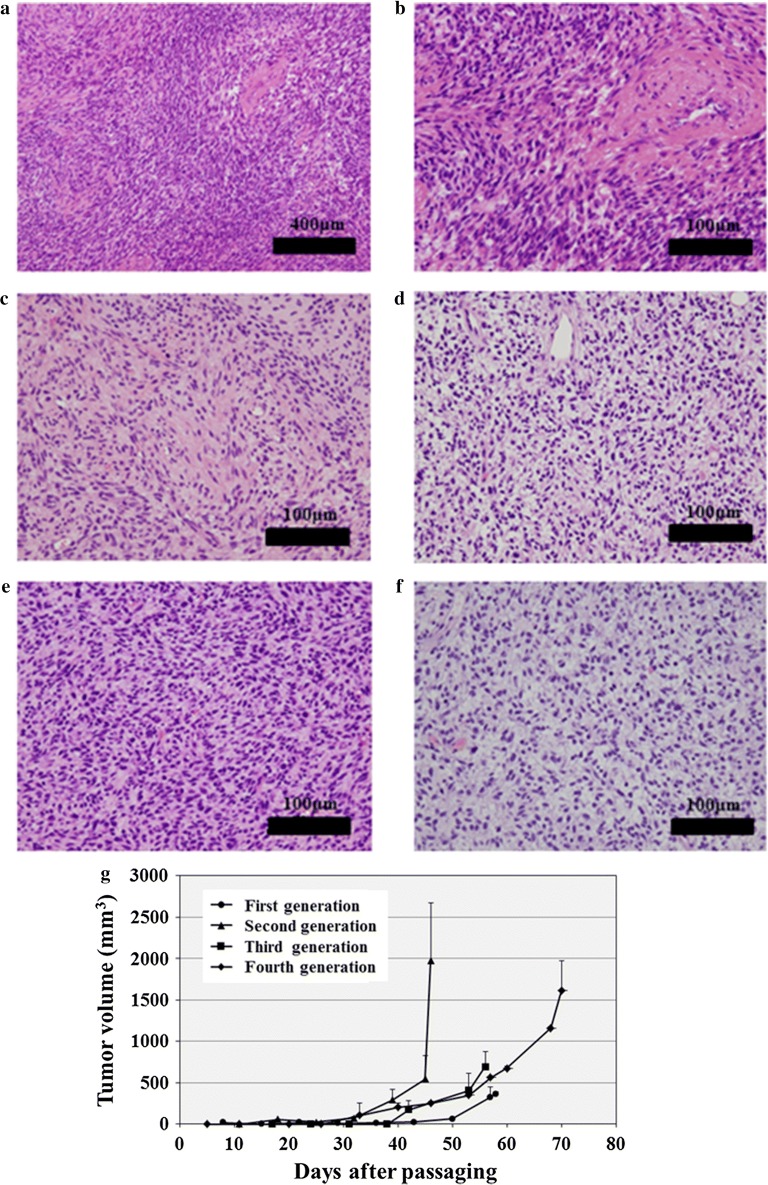
Histological analysis and growth curves of xenograft tumors derived from surgical specimens. **a**, **b** HE staining of original tumor tissue of the patient (Case 3). Original magnification: ×100 (**a**) and ×400 (**b**). **c**–**f** HE staining of the first- (**c**), second- (**d**), and third- (**e**) generation xenograft tissues derived from surgically resected samples. The fourth-generation xenograft is represented by the third-generation tumor that was frozen and re-implanted (**f**). Original magnification: ×400. **g** Growth of xenograft tumors, which started to increase in size at around 30 days after transplantation

Surgically resected tumor tissues were subcutaneously inoculated into immunodeficient mice; after in vivo propagation, they were serially transplanted twice. HE staining of tumors from different PDX transplantation passages indicated histopathological characteristics that were consistent with those of their original tumors (Fig. 3a, b). The xenograft tumor tissues at distinct passages exhibited histological characteristics of MPNST, i.e., highly cellular spindle-cell neoplasm differentiating to show elements of the nerve sheath, Schwann cells, and perineural cells (Fig. 3c–f). The tumors started to increase in size 30–50 days after transplantation (Fig. 3g); among them, the second-generation PDX tumors (NCC-MPNST3-X2) showed the most rapid growth and were used to establish a cell line (NCC-MPNST3-X2-C1). Furthermore, the growth of the third-generation xenograft tissue, which was frozen and later inoculated into mice, confirmed successful tumor propagation after prolonged storage (fourth-generation, Fig. 3g).

### Authentication of the established cell lines

We determined the statuses of ten microsatellite STRs in the original tumor tissues, PDXs, and cell lines (Table 2). All the established cell lines displayed distinct STR patterns, which were identical to those of the original primary tumor tissues, derived PDX tumors, and cell lines for each case, except for the TH01 loci and TPOX (2) loci in Case 1. A database search revealed that the obtained STR patterns differed from those of any cell line previously deposited in public cell banks such as ATCC, DSMZ, and JCRB, confirming that the established PDXs and cell lines were novel. Furthermore, we assessed the presence of Mycoplasma DNA in our cell lines using PCR and found no evidence of contamination (data not shown).

**Table 2:** Short tandem repeat analysis

Microsatellite (chromosome)	NCC-MPNST1-C1	Tumor tissue (Case 1)	NCC-MPNST2-C1	Tumor tissue (Case 2)
Amelogenin (X Y)	X, X	X, X	X, Y	X, Y
TH01 (3)	6	6, 9	9	9
D21S11 (21)	30, 31	30, 31	31, 2	31, 2
D5S818 (5)	7, 13	7, 13	11	11
D13S317 (13)	11, 12	11, 12	10	10
D7S820 (7)	10.3, 11	10.3, 11	8	8
D16S539 (16)	10, 11	10, 11	9, 11	9, 11
CSF1PO (5)	12	12	10, 12	10, 12
vWA (12)	17, 18	17, 18	15, 18	15, 18
TPOX (2)	11	8.11	11	11

Microsatellite (chromosome)	NCC-MPNST3-C1	NCC-MPNST3-X2-C1	NCC-MPNST3-X2	Tumor tissue (Case 3)
Amelogenin (X Y)	X, Y	X, Y	X, Y	X, Y
TH01 (3)	9	9	9	9
D21S11 (21)	30	30	30	30
D5S818 (5)	12, 13	12, 13	12, 13	12, 13
D13S317 (13)	8	8	8	8
D7S820 (7)	12, 13	12, 13	12, 13	12, 13
D16S539 (16)	11	11	11	11
CSF1PO (5)	12	12	12	12
vWA (12)	16	16	16	16
TPOX (2)	8, 11	8, 11	8, 11	8, 11

Microsatellite (chromosome)	NCC-MPNST4-C1	Tumor tissue (Case 4)	NCC-MPNST5-C1	Tumor tissue (Case 5)
Amelogenin (X Y)	X, X	X, Y	X	X, Y
TH01 (3)	6	6, 9	6, 7	6, 7
D21S11 (21)	30	30	31, 32.2	31, 33.2
D5S818 (5)	12, 12	12, 12	11	11
D13S317 (13)	10	10	8, 9	8, 9
D7S820 (7)	11	11	8, 12	8, 12
D16S539 (16)	9	9	10, 12	10, 12
CSF1PO (5)	12	12	11	11
vWA (12)	17	17	16, 17	16, 17
TPOX (2)	8	8	9, 11	9, 11

### Characterization of cell behavior

**Fig. 4:**
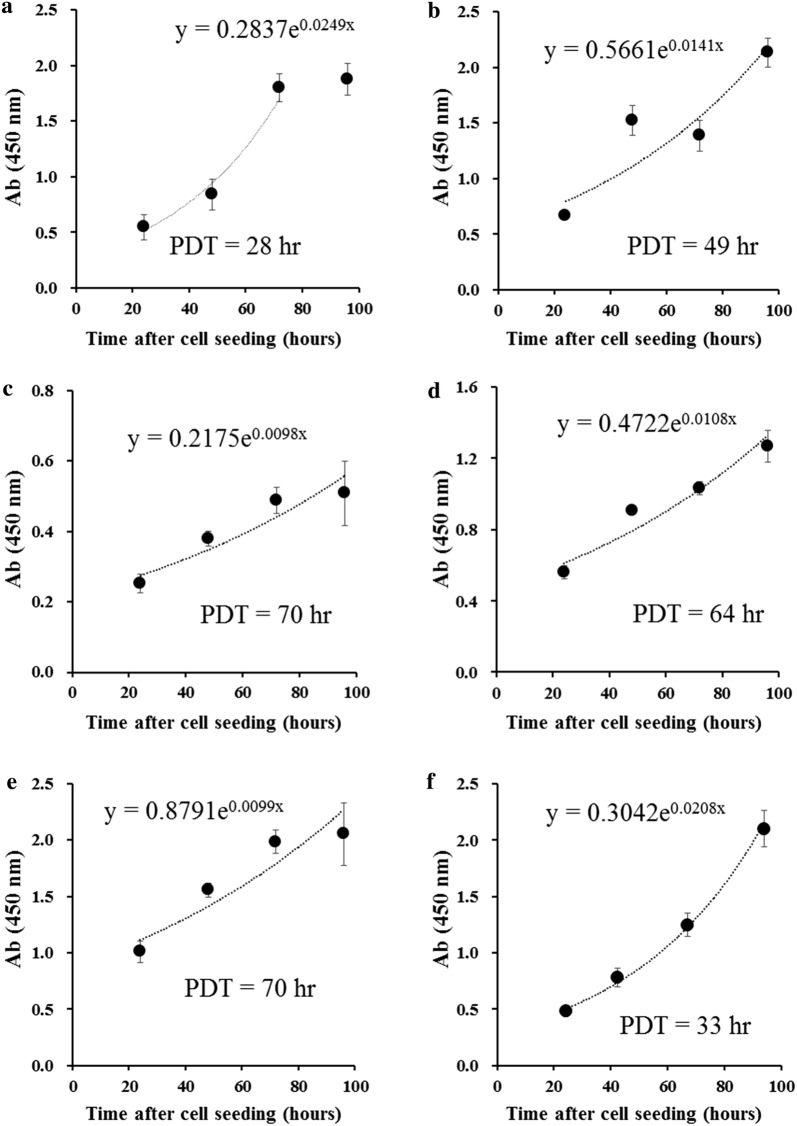
Proliferation of the established cell lines. Cell proliferation was evaluated using the CCK-8 assay. The numbers in the panel indicate the initial cell density at seeding (cells per well). **a** NCC-MPNST1-C1, **b** NCC-MPNST2-C1, **c** NCC-MPNST3-C1, **d** NCC-MPNST3-X2-C1, **e** NCC-MPNST4-C1, and **f** NCC-MPNST5-C1. *PDT* population doubling time

The growth rates of the cell lines were assessed by seeding a different number of cells and calculating the doubling time, which was 40, 57, 66, 58, 77, and 33 h for NCC-MPNST1-C1, NCC-MPNST2-C1, NCC-MPNST3-C1, NCC-MPNST3-X2-C1, NCC-MPNST4-C1, and NCC-MPNST5-C1 cells, respectively (Fig. 4).

**Fig. 5:**
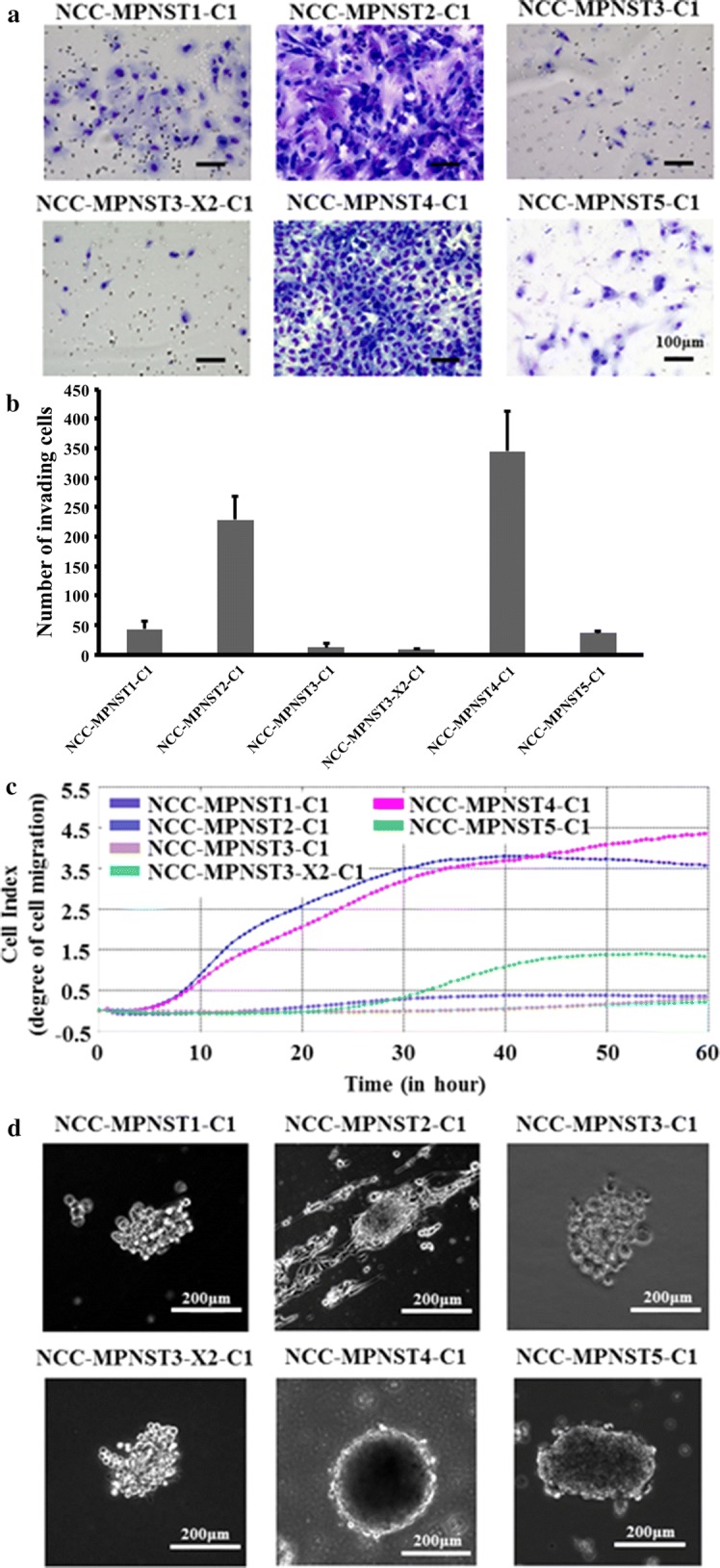
Invasive propensity and spheroid formation in the established cell lines. **a** Representative images of the Transwell invasion assay in the six cell lines. Scale bar: 100 μm. **b** Quantitative analysis of cell invasion, demonstrating differences in the invasive properties among the cell lines. **c** Invasion ability of cell lines measured by RTCA invasion assay. The y-axis indicates the degree of cell migration. **d** Spheroid formation in the five cell lines. The cells exhibited anchorage-independent growth on soft agar. Scale bar: 200 μm

The in vitro invasion assays revealed that the established cell lines exhibited different invasive abilities (Fig. 5a, b). The invasive potential of NCC-MPNST2-C1 and NCC-MPNST4-C1 cells appeared to be considerably strong, whereas that of NCC-MPNST1-C1, NCC-MPNST3-C1, and NCC-MPNST3-X2-C1 was rather weak. We observed consistent results with the conventional invasion assay in the RTCA invasion assay (Fig. 5c); two cell lines, NCC-MPNST2-C1 and NCC-MPNST4-C1, had higher invasion ability than the other four cell lines. We also evaluated the ability of the established cell lines to form spheroids, which are used in cancer research as in vitro three-dimensional tissue micro-analogs [39]. We observed that all cells formed spheroids when seeded on low attachment dishes (Fig. 5d).

### Genomic background of cell lines

We examined the status of genetic mutations in the established cell lines using NCC Oncopanel, which consists of the examinations of 114 actionable genes (Additional files 3, 4, 5, 6, 7, 8: Tables S3, S4, S5, S6, S7, S8). We found that the individual cell lines had a unique genetic mutation spectrum (Table 3), which may suggest the diverse response to treatments with anti-cancer agents. All tumor tissues except that from Case 1 had NF1 mutations. The mutations were present in CDKN2A in all cell lines except for NCC-MPNST2-C1 cells.

**Table 3:** Mutations in the established cell lines by NCC Oncopanel

Cell lines	Gene	Alterations	Protein effect	Database	
				COSMIC ID	ClinVar ID
NCC-MPNST1-C1	CDKN2A	Homozygous deletion	No expression	–	–
	TP53	Homozygous deletion	No expression	–	–
	TSC1	Homozygous deletion	No expression	–	–
	BRCA2	NM_000059.3:c.5614A>T	p.K1872*	Not available	RCV000031560
NCC-MPNST2-C1	NF1	NM_001042492.2:c.536_539del	p.L179Yfs*11	Not available	Not available
NCC-MPNST3-C1	CDKN2A	Homozygous deletion	No expression	–	–
	MTOR	Homozygous deletion	No expression	–	–
	PIK3CA	NM_006218.4:c.1624G>A	p.E542K	COSM760	RCV000024622
	NF1	NM_001042492.2:c.2446C>T	p.R816*	COSM24444	Not available
NCC-MPNST3-X2-C1	CDKN2A	Homozygous deletion	No expression	–	–
	PIK3CA	NM_006218.4:c.1624G>A	p.E542K	COSM760	RCV000024622
	NF1	NM_001042492.2:c.2446C>T	p.R816*	COSM24444	Not available
	TP53	NM_001126115.1:c.9C>G	p.C3W	COSM44219	Not available
NCC-MPNST4-C1	CDKN2A	Homozygous deletion	No expression	–	–
	KDM6A	Homozygous deletion	No expression	–	–
	ARAF	Homozygous deletion	No expression	–	–
	TP53	NM_001126112.2:c.745A>G	p.R249G	COSM10668	Not available
	NF1	NM_001042492.2:c.5812 + 3delAGTA	Splice site	Not available	Not available
NCC-MPNST5-C1	CDKN2A	Homozygous deletion	No expression	–	–
	NF1	NM_001042492.2:c.1756_1759del	T586Vfs*18	COSM6853566	Not available

ARAF, A-Raf proto-oncogene, serine/threonine kinase; BRCA2, BRCA2 (breast cancer 2) DNA repair associated; CDKN2A, cyclin dependent kinase inhibitor 2A; KDM6A, lysine demethylase 6A; MTOR, mechanistic target of rapamycin kinase; NF1, neurofibromin 1; PIK3CA, phosphatidylinositol-4,5-bisphosphate 3-kinase catalytic subunit alpha; TP53, tumor protein p53; TSC1, TSC (tuberous sclerosis 1) complex subunit 1

### Sensitivity of cell lines to anti-cancer drugs

**Fig. 6:**
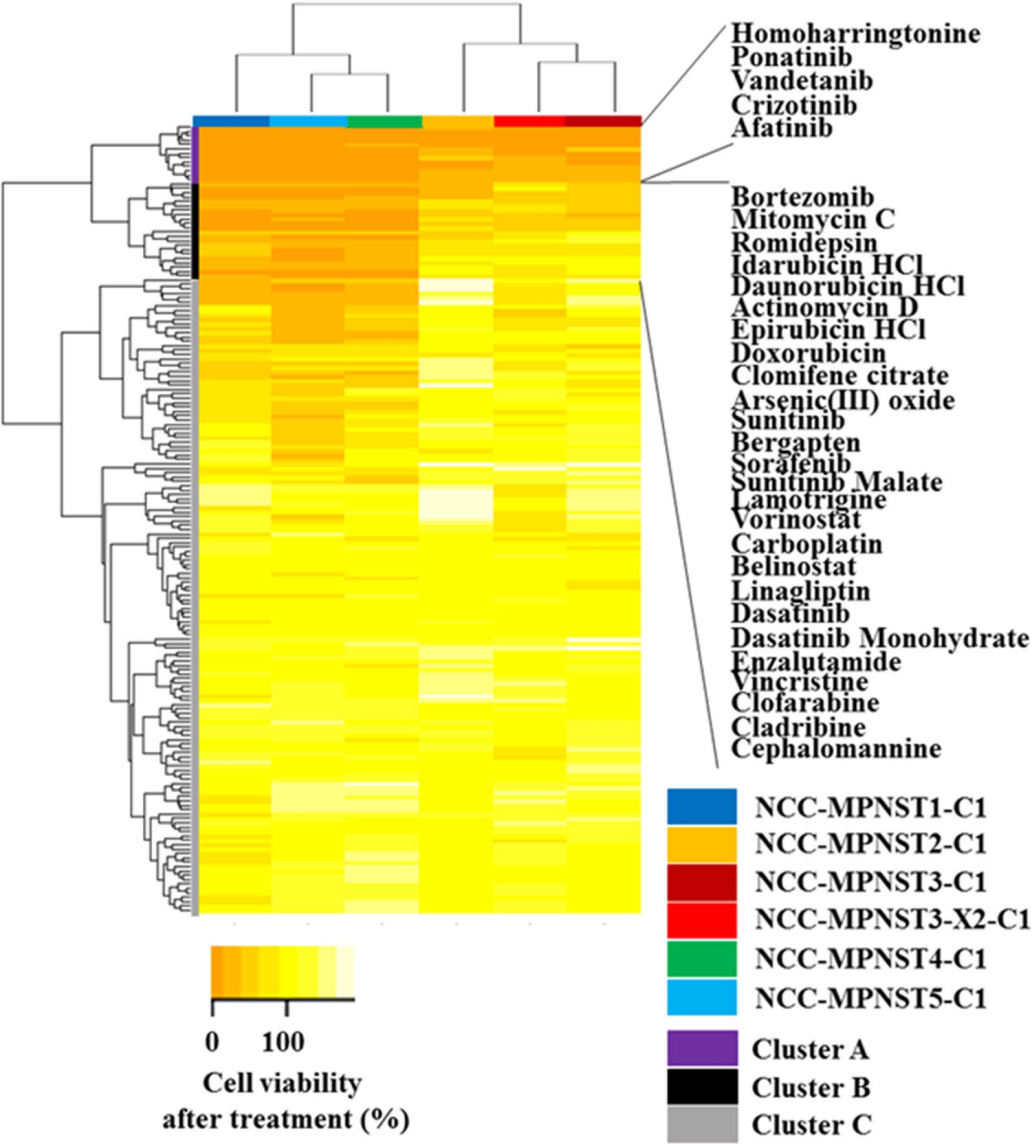
Hierarchical analysis of cell lines and anti-cancer drugs. The cell lines and anti-cancer drugs were grouped based on the growth suppression data (Additional file 3: Table S3) using hierarchical cluster analysis. The cells were treated with 10 µM of each drug and analyzed for viability, which was calculated after comparison with the control and presented as a heat map. The names of the cell lines and anti-cancer drugs are color-coded

Finally, we assessed the sensitivity of the established cell lines to treatment with known chemotherapeutic agents and quantitatively evaluated cell viability (Additional file 9: Table S9). Hierarchical cluster analysis of the cell lines and anti-cancer drugs based on the growth suppression data suggested that the established MPNST cells showed unique drug response patterns (Fig. 6). Chemotherapeutic agents in cluster A exerted anti-proliferative effects on all five cell lines. All anti-cancer drugs in cluster B showed growth-suppressive effects on NCC-MPNST1-C1, NCC-MPNST4-C1, and NCC-MPNST5-C1 cells, which clustered together. The agents in cluster C did not show significant effects on the examined cell lines.

## Discussion

Patient-derived cancer models are useful tools not only for basic research but also for preclinical studies. Various cell lines obtained from donors with different clinical background possess distinct in vitro characteristics, thereby representing a unique tool for clinical research. In addition, the patient-derived cell lines may retain original characteristics of the tumor tissues, making them adequate resources for the study of disease etiology and tumor cell origin. Here, we describe the establishment of six cell lines and one PDX from tumor tissues of five MPNST patients with different clinicopathological features. These cell lines may reflect the clinical diversity of the disease and can be used to further our understanding of various MPNST manifestations. The comparative study between the features of the tumor in patients and the characters of cell lines and the PDX is intriguing. To perform such studies, we need enough number of patient-derived cancer models, considering the diversity of the disease.

The clinical backgrounds and characteristics of the cell lines used in this study were diverse. Cases 1, 3, and 5 were diagnosed with NF1, however, Cases 2 and 4 were not, and the tumor in Case 1 originated in the bone [38], whereas tumors in the other cases developed from soft tissue. The growth of tumor cell lines established from these patients showed significant differences. The cell lines established from Cases 1 and 5 (NCC-MPNST1-C1 and NCC-MPNST5-C1, respectively) exhibited relatively short population doubling time, whereas those from Cases 3 and 4 (NCC-MPNST3-C1 and NCC-MPNST4-C1, respectively) showed the longest doubling time. The propensity for invasion also differed considerably: the cell lines established from Cases 2 and 4 (NCC-MPNST2-C1 and NCC-MPNST4-C1, respectively) showed remarkable invasive potential, whereas those from Cases 1 (NCC-MPNST1-C1), and 3 (NCC-MPNST3-C1 and NCC-MPNST3-X2-C1), and 5 (NCC-MPNST5-C1) had a noticeably lower invasive ability. These observations may suggest that tumor cells from patients with NF1 were less invasive. However, the results were controversial; the genomic study indicated that the cell lines except NCC-MPNST1-C1 had a mutation in NF1. In the previous reports, the association of poor MPNST prognosis with NF1 is also highly controversial; although studies have shown that MPNST patients with NF1 had poorer prognosis than those without NF1 [40], meta-analyses of more than 1,800 patients with MPNST suggested that their survival did not depend on the presence of NF1 [41]. Further, Cases 3 and 5 had metastases, and the lower invasion potential of the corresponding cell lines seemed to be inconsistent with the clinical observations. The association between the response to anti-cancer agents and the genomic backgrounds is complex, in part due to the limited number of cases analyzed in the study. Although the associations between cell line characteristics and the clinicopathological and genomic backgrounds of donor patients are hypothetical at this stage, it can be beneficial for further studies on MPNSTs, and they should be further investigated using a significantly larger number of patients. In addition, it is also worth investigating whether the established cell lines represented the overall features of the original tumors. Although we did not clone the cell line, the tumor cells which adapted themselves to the tissue culture condition may be selectively expanded. This possibility should be further considered when using the cell line. Overall, due to their diverse characteristics, our cell lines can act as useful resources for addressing this issue using in vitro experiments.

We observed that the proliferation potential of tumor cells in PDX models varied and depended on the passage number (Fig. 3). Thus, optimization of patient-derived cancer models is required to predict the response to treatment in PDX. We observed that all established cell lines harbored the potential to form spheroids. Several lines of evidence suggested the utility of spheroids for drug screening assay [42, 43]. Hence, the functional properties of spheroids derived from the established cell lines warrant further investigations.

The responses of the established cell lines to treatment with anti-cancer agents were also intriguing. According to their sensitivity to the agents, cell lines were classified into two groups; the first consisted of NCC-MPNST1-C1, NCC-MPNST4-C1, and NCC-MPNST5-C1, and the other of NCC-MPNST2-C1, NCC-MPNST3-C1, and NCC-MPNST3-X2-X1. The examined anti-cancer agents were also grouped into two categories according to the effects on the MPNST cells, such as those in Cluster A and Cluster B. The molecular backgrounds of MPNST cells about the sensitivity to anti-cancer agents are worthy of further investigation.

The present study had certain apparent limitations. First, the number of examined cases and established cell lines used in this study were small. Hence, the observed correlation trends between clinical characteristics and cell behavior in vitro should be validated using more patients and cell lines. The general question of how faithfully the established patient-derived in vitro*/*in vivo models can predict the clinical response of MPNST patients should be addressed using a statistically adequate number of cases in the prospective studies. Second, the molecular mechanisms underlying the described in vitro observations should be investigated to confirm the utility of patient-derived cancer models using comprehensive experimental approaches such as genomics, proteomics, and proteogenomics. Third, the further characterization of established cell lines should be required to establish the similarities between the cell lines and original tumor cells. Further, many factors can affect cell line characteristics, therefore, it is worth investigating the typical genetic and epigenetic aberrations of MPNST, including *NF1* gene mutations [16], and loss of histone H3K27 methylation [12], which is attributable to EED and SUZ12 alterations. Finally, since tumor cells are selected under the artificial pressures of tissue culture conditions, and considering that MPNST exhibits heterogeneous histology, the tumor sections examined for microscopic pathological diagnosis may not be identical those used for cell line establishment. Thus, the origin of established cell lines is of great interest, and worth investigating, in terms of etiology of disease. Hence, it is imperative to evaluate the similarity between the original tumors, PDXs, and cell lines to use them appropriately.

## Conclusions

We successfully established PDXs and cell line models of MPNST with different genomic backgrounds and in vitro characteristics, such as drug responses. As our cell lines exhibited different phenotypes, probably reflecting those of their original tumors, they will be useful research tools for studying the clinical diversity of MPNST. Continuous efforts for developing and analyzing patient-derived cancer models will contribute to the improvement of clinical trials and the development of effective therapies for MPNST and other cancers.

## Supplementary information

**Additional file 1: Table S1**. Genes examined by NCC Oncopanel.

**Additional file 2: Table S2.** Anti-cancer agents examined in this study.

**Additional file 3: Table S3.** Summary of detections by NCC Oncopanel.

**Additional file 4: Table S4.** Detection of SNV and indels by NCC Oncopanel.

**Additional file 5: Table S5.** Detection of CNV by NCC Oncopanel.

**Additional file 6: Table S6.** Detection of fusion by NCC Oncopanel.

**Additional file 7: Table S7.** Detection of Mapping by NCC Oncopanel.

**Additional file 8: Table S8.** Detection of depth by NCC Oncopanel.

**Additional file 9: Table S9.** Cell viability after treatment with anti-cancer agents.

## Data Availability

All data and the cell lines are available upon a request. As PDX is a limited resource, it will be provided under the appropriate conditions.

## Abbreviations

ATCC: American Type Culture Collection
DMEM: Dulbecco’s modified Eagle’s medium
DSMZ: Deutsche Sammlung von Mikroorganismen und Zellkulturen
HE: Hematoxylin and eosin
JCRB: Japanese Collection of Research Bioresources
MRI: Magnetic resonance imaging
MPNST: Malignant peripheral nerve sheath tumor
NGS: Next-generation sequencing
PDX: Patient-derived xenograft
RTCA: Real-time cell analyzer
SNV: Single nucleotide variants
STR: Short tandem repeat
